# Editorial: Advancing collaborative efforts in cancer research: a convening of north-eastern Nigeria universities and international health systems on etiology, care, and outcomes

**DOI:** 10.3389/fonc.2025.1611783

**Published:** 2025-05-06

**Authors:** Musa Abubakar Garbati, Sophia H. L. George, Aisha Mustapha, Bala Mohammed Audu, Matthew Schlumbrecht

**Affiliations:** ^1^ Department of Medicine, Federal University of Health Sciences, Azare, Bauchi, Nigeria; ^2^ Division of Gynecologic Oncology, Department of Obstetrics, Gynecology and Reproductive Sciences, Sylvester Comprehensive Cancer Center, University of Miami Miller School of Medicine, Miami, FL, United States; ^3^ Department of Obstetrics and Gynecology, Ahmadu Bello University Teaching Hospital, Zaria, Kaduna, Nigeria; ^4^ Department of Obstetrics and Gynecology, Federal University of Health Sciences, Azare, Bauchi, Nigeria

**Keywords:** Nigeria, cancer equity, global health, awareness, capacity building

In northeastern Nigeria, a region marked by limited healthcare resources, socioeconomic challenges, and cultural barriers, the burden of cancer is significant (1). Recent studies have highlighted the need for multidisciplinary collaboration and systemic interventions to improve cancer care outcomes (2). This editorial synthesizes the findings of six key papers that explore various aspects of cancer management in northeastern Nigeria, emphasizing the need for a holistic approach to address this growing crisis.

## Mental health in oncology care

*“ The Role of a Mental Health Physician in the Management of Oncology Patients: A Case Vignette and the Need for Collaboration”* underscores the critical role that mental health professionals play in supporting cancer patients (Armiya’u and Akande). The study used case vignettes to illustrate how mental health physicians can address these challenges through personalized interventions, such as psychotherapy, medication management, and coping strategies. The article emphasizes that mental health care should be integrated into standard oncology practice and that stronger partnerships between oncology and mental health teams should be developed to provide comprehensive care.

## The burden of hematologic malignancies

*“Common Hematological Malignancies in Northeastern Nigeria: A Multi-Centre Study of Their Pattern, Distribution, and Treatment Challenges”* investigates the prevalence, types, and obstacles to treatment of blood cancers in northeastern Nigeria (Dachi et al.). The study, conducted across multiple healthcare centers, identifies non-Hodgkin lymphoma, leukemia, and multiple myeloma as the leading hematologic malignancies in the region. The study highlights significant challenges in the management of these malignancies, including limited diagnostic facilities, inadequate treatment options, and poor access to specialized care, which are exacerbated by financial constraints and lack of awareness. The findings underscore the need for improved healthcare infrastructure and enhanced public awareness. This contribution calls for a coordinated effort to improve early diagnosis and expand treatment options.

## The burden of cancer in rural Sub-Saharan Africa

*“Emerging Cancer Disease Burden in a Rural Sub-Saharan African Population: Northeast Nigeria in Focus”* examines the increasing prevalence of cancer in a rural, underserved region of sub-Saharan Africa (Ezenkwa et al.). Common cancer types identified include those of the breast, cervical, spine, prostate, and liver, which are often diagnosed at advanced stages due to delays in seeking medical attention. The study emphasizes the socio-economic and cultural barriers that hinder effective cancer management, including poverty, stigma, lack of access to healthcare facilities, and reliance on traditional medicine. It also points to the region’s lack of oncology specialists, diagnostic tools, and treatment facilities. The authors emphasize the need for collaboration among governments, non-governmental organizations, and international partners to reduce the cancer burden and improve outcomes for rural populations.

## Gynecologic malignancies: a growing concern

*“The Spectrum of Gynecologic Malignancies in Northeastern Nigeria”* investigates the prevalence, types, and characteristics of gynecologic cancers in this territory (Katagum et al.). The study identifies cervical cancer as the most common gynecologic malignancy in the region, followed by ovarian and uterine cancers, while also highlighting the challenges faced in the management of gynecologic cancers in this resource-limited setting, including inadequate diagnostic facilities, a shortage of skilled healthcare professionals, a lack of formalized screening programs, and low symptom awareness. Cultural factors and stigma also contribute to delays in seeking medical care. The authors advocate for targeted interventions, such as HPV vaccination programs and capacity building for healthcare providers, to reduce incidence and improve outcomes for women with gynecologic cancers in the region.

## Cervical cancer awareness

*“Cervical Cancer Awareness, Perception, and Attitude Among Tertiary Health Institution Students in Northeastern Nigeria”* explores the level of knowledge, along with perceptions and attitudes, toward cervical cancer among university students (Muhammad et al.). The study reveals that while a significant proportion are aware of cervical cancer, their understanding of its causes, risk factors, and prevention methods, such as HPV vaccination and regular screening, is limited. Misconceptions and knowledge gaps were particularly evident regarding the role of HPV infection and the importance of early detection. This contribution highlights cultural and educational barriers that contribute to low awareness, including stigma, lack of health education programs, and insufficient emphasis on reproductive health in the school curriculum. However, the study also found that students generally express positive attitudes toward learning more about cervical cancer and participating in prevention efforts. The article calls for comprehensive educational campaigns and the integration of cervical cancer awareness programs into university health curricula.

## Pediatric cancers

*“Incidence of Childhood Cancers in the North East Geopolitical Zone of Nigeria”* examines the prevalence of childhood cancers in northeastern Nigeria (Suleiman et al.). The study identifies a significant burden of pediatric malignancies in the region, including lymphoma, leukemia, and retinoblastoma. These cancers predominantly affect children under the age of 10, with variations in incidence based on age, gender, and geographic location. The study highlights the challenges of diagnosing and managing childhood cancers in this resource-limited setting, including inadequate healthcare infrastructure, lack of specialized pediatric oncology services, and limited access to diagnostic tools and treatment options. The authors advocate for the establishment of specialized treatment centers, training programs for healthcare providers, and community-based awareness campaigns to promote early detection and improve survival rates for affected children.

## A way forward: recommendations for action

The findings from these studies paint a stark picture of the cancer burden in northeastern Nigeria, but they also provide a roadmap for action. Key recommendations include:

1. Strengthening Healthcare Infrastructure: Establishing specialized cancer treatment centers and improving access to diagnostic tools and therapies.
2. Enhancing Public Health Education: Launching targeted awareness campaigns to promote early detection, HPV vaccination, and regular screening.
3. Integrating Mental Health Services: Incorporating mental health care into standard oncology practice to address the psychological burden of cancer.
4. Capacity Building: Training healthcare providers in oncology and mental health care to improve service delivery.
5. Community Engagement: Empowering communities through education and outreach programs to reduce stigma and promote early medical consultation.
6. Policy and Funding Support: Increasing funding for cancer care and advocating for policies that prioritize cancer prevention and treatment in the national health agenda.

## Conclusion

The findings from these studies serve as a call to action for policymakers, healthcare providers, and the global health community to prioritize cancer care in northeastern Nigeria and similar settings. Only through collective efforts can we hope to achieve equitable cancer care for all.
